# Genetic and Cellular Architecture of Breast Cancer Risk in Multi-Ancestry Studies of 159,297 Cases and 212,102 Controls

**DOI:** 10.1101/2025.08.20.25334075

**Published:** 2025-11-07

**Authors:** James L. Li, Maria Zanti, Jacob Williams, Om Jahagirdar, Guochong Jia, Alistair Turcan, Qiang Hu, Jean-Tristan Brandenburg, Li Yan, Weang-Kee Ho, Jingmei Li, José Patricio Miranda, Devika Godbole, Julie-Alexia Dias, Xiaomeng Zhang, Leila Dorling, Wenlong Carl Chen, Nicholas Boddicker, Ying Wang, Alicia Martin, Yan Dora Zhang, Joe Dennis, Esther M. John, Gabriela Torres-Mejia, Larry Kushi, Jeffrey Weitzel, Susan L. Neuhausen, Luis Carvajal-Carmona, Christopher Haiman, Elad Ziv, Laura Fejerman, Wei Zheng, Dezheng Huo, Douglas Easton, Stephen J. Chanock, Nilanjan Chatterjee, Peter Kraft, Montserrat Garcia-Closas, Wendy S.W. Wong, Kyriaki Michailidou, Qianqian Zhu, Martin Jinye Zhang, Diptavo Dutta, Thomas U. Ahearn, Haoyu Zhang

**Affiliations:** 1 Department of Public Health Sciences, University of Chicago, Chicago, IL, USA; 2 Biostatistics Unit, The Cyprus Institute of Neurology and Genetics, Nicosia, Cyprus; 3 Division of Cancer Epidemiology and Genetics, National Cancer Institute, National Institutes of Health, Bethesda, MD, USA; 4 Division of Epidemiology, Department of Medicine, Vanderbilt Epidemiology Center, Vanderbilt-Ingram Cancer Center, Vanderbilt University Medical Center, Nashville, TN, USA; 5 Ray and Stephanie Lane Computational Biology Department, School of Computer Science, Carnegie Mellon University, Pittsburgh, PA, USA; 6 Department of Biostatistics and Bioinformatics, Roswell Park Comprehensive Cancer Center, Buffalo, NY, USA; 7 Sydney Brenner Institute for Molecular Bioscience, Faculty of Health Sciences, University of the Witwatersrand, Johannesburg, South Africa; 8 Strengthening Oncology Services Research Unit, Faculty of Health Sciences, University of the Witwatersrand, Johannesburg, South Africa; 9 School of Mathematical Sciences, Faculty of Science and Engineering, University of Nottingham Malaysia, Semenyih, Selangor, Malaysia; 10 Cancer Research Malaysia, Subang Jaya, Selangor, Malaysia; 11 Genome Institute of Singapore, Agency for Science, Technology and Research (A*STAR), Singapore; 12 Department of Nutrition, Diabetes and Metabolism, School of Medicine, Pontificia Universidad Católica de Chile, Santiago, Chile; 13 PhD Program in Epidemiology, Pontificia Universidad Católica de Chile, Santiago, Chile; 14 Advanced Center for Chronic Diseases, Pontificia Universidad Católica de Chile & Universidad de Chile, Santiago, Chile; 15 Department of Biostatistics, Harvard T.H. Chan School of Public Health, Boston, MA, USA; 16 Centre for Cancer Genetic Epidemiology, Department of Public Health and Primary Care, University of Cambridge, Cambridge, UK; 17 Department of Quantitative Health Sciences, Mayo Clinic, Rochester, MN, USA; 18 Analytic and Translational Genetics Unit, Massachusetts General Hospital, Boston, MA, USA; 19 Department of Medicine, Harvard Medical School, Boston, MA, USA; 20 Stanley Center for Psychiatric Research, Broad Institute of MIT and Harvard, Cambridge, MA, USA; 21 Department of Statistics & Actuarial Science, School of Computing and Data Science, The University of Hong Kong, Hong Kong SAR, China; 22 Departments of Epidemiology & Population Health and of Medicine (Oncology), Stanford University School of Medicine, Stanford, CA, USA; 23 Stanford Cancer Institute, Stanford University School of Medicine, Stanford, CA, USA; 24 Instituto Nacional de Salud Pública, Cuernavaca, Mexico; 25 UC Davis Genome Center, University of California, Davis, Davis, CA, USA; 26 Division of Research, Kaiser Permanente Northern California, Oakland, CA, USA; 27 Division of Precision Prevention, University of Kansas Comprehensive Cancer Center, Kansas City, KS, USA; 28 Department of Population Sciences, Beckman Research Institute of City of Hope, Duarte, CA, USA; 29 Department of Biochemistry and Molecular Medicine, School of Medicine, University of California at Davis, Davis, CA, USA; 30 The Health Equity, Leadership, Science and Community Laboratory, Genome Center, University of California at Davis, Davis, CA, USA; 31 Department of Preventive Medicine, Norris Comprehensive Cancer Center, Keck School of Medicine, University of Southern California, Los Angeles, CA, USA; 32 Division of General Internal Medicine, Department of Medicine, University of California, San Francisco, San Francisco, CA, USA; 33 Department of Public Health Sciences, University of California Davis, Davis, CA, USA; 34 Genome Center, University of California Davis, Davis, CA, USA; 35 UC Davis Comprehensive Cancer Center, University of California Davis, Davis, CA, USA; 36 Centre for Cancer Genetic Epidemiology, Department of Oncology, University of Cambridge, Cambridge, UK; 37 Department of Biostatistics, Johns Hopkins University, Baltimore, MD, USA; 38 Department of Oncology, Johns Hopkins School of Medicine, Baltimore, MD, USA; 39 Cancer Epidemiology and Prevention Research Unit, The Institute of Cancer Research and Imperial College London, London, UK; 40 National Cancer Registry, a Division of the National Institute for Communicable Diseases, National Health Laboratory Service, Johannesburg, South Africa

## Abstract

Breast cancer genome-wide association studies (GWAS) have identified over 200 independent genome-wide significant susceptibility markers. However, most studies have focused on one or two ancestral groups. We examined breast cancer genetic architecture using GWAS summary statistics from African (AFR), East Asian (EAS), European (EUR) and Hispanic/Latina (H/L) samples, totaling 159,297 cases and 212,102 controls, comprising the largest multi-ancestry study of breast cancer to date. The logit-scale heritability of breast cancer ranged from $h^{2}=0.47$ (SE = 0.07) in EAS to AFR $h^{2}=0.61$ (SE = 0.10), with no significant differences across ancestries (p=0.63). The estimated number of susceptibility markers in a sparse normal-mixture effects model also varied from 4,446 (SE = 3,100) in EAS to 8,308 (SE = 2,751) in AFR, but differences were not significant across ancestries (p=0.55). Cross-sample genetic correlations varied, with the strongest correlation between EUR and EAS (*ρ* = 0.79, SE = 0.08) and weakest between AFR and H/L (*ρ* = 0.26, SE = 0.24). Common variants in regulatory elements were enriched for genetic association across samples. By integrating the GWAS summary statistics with the Tabula Sapiens scRNA-seq atlas, we identified ancestry-shared associations between breast cancer and specific cell types, including innate immune cells, secretory epithelial cells and stromal cells. Collectively, these results support a largely shared polygenic architecture of breast cancer across ancestries, with consistent enrichment of common regulatory variants and convergent cellular signatures identified through single-cell analyses.

## INTRODUCTION

Breast cancer is the most common cancer among women worldwide, affecting over two million individuals annually^1^. Genome-wide association studies (GWAS) have identified more than 200 genome-wide significant susceptibility markers associated with breast cancer risk^2–7^. However, most GWAS have focused on populations of European (EUR) and East Asian (EAS) ancestry samples^2,4–9^, while studies in African (AFR) ^3,10–13^ and Hispanic/Latina (H/L)^14,15^ samples are limited, and often with smaller sample sizes. Existing studies have both replicated risk markers identified across ancestry populations, as well as identified population-specific risk markers. Furthermore, polygenic risk score (PRS) models trained in European samples often exhibit lower predictive performance in other genetic ancestry samples^16^, emphasizing the need for a broader understanding of breast cancer genetics across different ancestries.

We examined the genetic architecture of breast cancer using GWAS summary statistics from a total of 159,297 cases and 212,102 controls from AFR, EAS, EUR and H/L samples (Supplementary Figure 1, Supplementary Table 1). We estimated genome-wide single-nucleotide polymorphism (SNP)-based heritability, the number of susceptibility genetic markers, and assessed genetic correlations across samples. We further investigated enrichment of regulatory elements associated with breast cancer risk, and applied the single-cell disease-relevance score (scDRS+) method to identify cell types implicated in breast cancer across ancestry samples. These findings further refine our understanding of breast cancer genetics and highlight the influence of common variants and regulatory elements across ancestries.

## RESULTS

### Logit-scale SNP-based heritability

We used linkage-disequilibrium (LD) score regression^17^ to estimate the logit-scale SNP-based heritability $\left(h^{2}\right)$, which quantifies the proportion of variance in the log-odds of disease explained by common genetic variants under a logistic regression model (Methods). To obtain a robust estimate of the heritability, we conducted sensitivity analyses by applying various SNP inclusion criteria, including different minor allele frequency thresholds (MAF > 0.01 or MAF > 0.05), and SNP arrays (HapMap3^18^ or HapMap3 + Multi-Ethnic Genotyping Array (MEGA)^19^). LD scores were computed using 1000 Genomes Project (1000G)^20^ Phase 3 for AFR, EAS and EUR samples, and Admixed American (AMR) as reference for H/L samples. For AFR, we additionally generated estimates using a subset of 2,731 controls from the African Ancestry Breast Cancer Genetics (AABCG) Consortium.

Heritability estimates for EUR and EAS samples remained consistent across different SNP inclusion criteria hat varied by minor allele frequency thresholds (MAF > 0.01 vs. MAF > 0.05) and SNP sets (HapMap3 vs. HapMap3 + MEGA) (Supplementary Table 2). Thus, we adopted the most comprehensive SNP set (MAF > 0.01 and HapMap3 plus MEGA SNPs) for our final analyses. In contrast, heritability estimates for AFR varied significantly depending on the LD reference panel ($h^{2}=0.27$, SE=0.11 to $h^{2}=0.61$, SE=0.10; Supplementary Table 2). Given that the GWAS summary statistics for AFR were primarily from U.S. samples (85.3%)^12^, while AFR in 1000G are mostly from African continental samples, AABCG likely provides more appropriate reference for LD estimation in this context. For H/L, heritability estimates were less stable due to the smaller GWAS sample size, leading to larger standard errors. To enhance robustness, we applied the more conservative MAF > 0.05 threshold.

Across ancestry samples, SNP-based heritability estimates ranged from EAS $h^{2}=0.466$ (SE=0.066) to AFR $h^{2}=0.614$ (SE=0.095), with EUR $h^{2}=0.501$ (SE=0.050) and H/L $h^{2}=0.588$ (SE=0.360) showing intermediate values (Table 1). While point estimates varied modestly, we found no statistically significant evidence of heterogeneity in SNP-based heritability across ancestry samples (Cochran’s Q = 1.72, p = 0.63, Supplementary Figure 2). Overall, these findings suggest common susceptibility variants moderately contribute to breast cancer risk across samples. The slightly higher point estimate in AFR could reflect the broader range of genetic variation captured by a larger set of well-imputed SNPs (n=1,628,778) (Table 1).

### The polygenicity of breast cancer

Breast cancer exhibits a high degree of polygenicity across populations. Using the GENESIS package^21,22^, we modeled the distribution of marker effect sizes for AFR, EAS and EUR samples as a sparse mixture of normal. Due to a limited sample size, the model for H/L did not converge, precluding a reliable estimate. Across the three analyzed samples, marker effect sizes showed no evidence of inflation ( Supplementary Figure 3). The estimated polygenicity (number of markers with non-zero effects) was highest in AFR (8,308, SE=2,751), followed by EUR (5,235, SE=1,191) and EAS (4,446, SE=3,100, Supplementary Table 3). We found no statistically significant heterogeneity in polygenicity across samples (Cochran’s Q = 1.20, p = 0.55), that observed differences are consistent with sampling variation. These findings support a broadly shared polygenic basis of breast cancer across ancestries, with modest differences that may reflect sample-specific power or LD structure.

**Figure 1.**
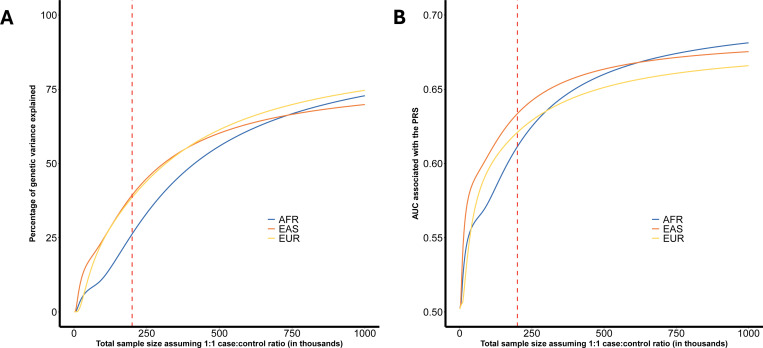
Projected genetic variance explained and predictive performance of clumping and thresholding (CT) polygenic risk scores (PRSs) across samples at varying GWAS sample sizes. A) Estimated proportion of GWAS heritability explained by CT PRS, as a function of total GWAS sample sizes, using the GENESIS framework. The proportion was calculated as the variance explained by the PRS divided by the logit-scale heritability of common variants. B) Projected area under the curve (AUC) for CT PRS models at different sample sizes. Detailed projection of explained genetic variance and AUC at different sample size are provided in Supplementary Table 4. The red dashed line indicates a total sample size of 200,000 individuals (100,000 cases and 100,000 controls). Abbreviations: AFR, African; EAS, East Asian; EUR, European.

We used GENESIS to project the proportion of heritability explained and the accuracy of polygenic risk scores (PRS) at increasing GWAS sample sizes (Figure 1A, Supplementary Table 4). Polygenic risk scores evaluated in GENESIS were based on the clumping and thresholding (CT-PRS) approach^23,24^, applied independently to each sample using ancestry-specific GWAS summary statistics and corresponding LD reference panels. The proportion of genetic variance explained was defined as the variance captured by the CT-PRS divided by the logit-scale heritability of common variants. At a GWAS sample size of 100,000 cases and 100,000 controls, the proportion of genetic variance explained was 26.2% in AFR, 39.4% in EAS, and 38.6% in EUR (Supplementary Table 4). Projected area under the curve (AUC) values for CT-PRS followed a similar trend. At a sample size of 100,000 cases and 100,000 controls, the expected AUC was 61.1% for AFR, 63.4% for EAS and 62.1% for EUR (Figure 1B, Supplementary Table 4). Asymptotically (i.e., at infinite sample size), the estimated maximum AUCs for CT-PRS were 71% for AFR, and 71% for EAS, and 69% for EUR (Supplementary Table 3).

### Cross-sample genetic correlations of breast cancer

**Figure 2.**
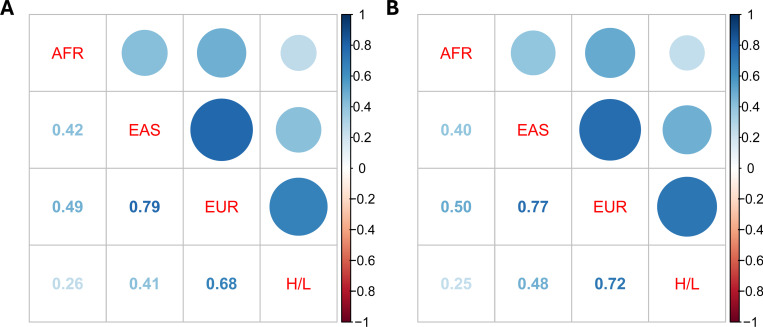
Heatmaps for cross-sample genetic correlations of breast cancer estimated by POPCORN. for A) HapMap3 variants and B) HapMap3 and Multi-Ethnic Genotyping Array variants combined. Note: African, AFR; Hispanic/Latina, H/L; East Asian, EAS; European, EUR.

We next assessed cross-sample genetic correlations of breast cancer using *Popcorn*^25^, which estimates genetic effect correlations accounting for LD differences across ancestries. Using variants from the HapMap3 panel, we identified the strongest genetic correlations between EUR and EAS (ρ: 0.79, SE: 0.08) and EUR and H/L (ρ: 0.68, SE: 0.21), indicating substantial shared genetic architecture between these samples (Figure 2A, Supplementary Table 5). In contrast, genetic correlations involving AFR were consistently lower, with estimates ranging from AFR and H/L (ρ = 0.26, SE = 0.24) to AFR and EUR (ρ = 0.42, SE = 0.14), suggesting greater divergence in effect sizes of variants tagging in AFR. We further evaluated whether expanding the variant set to include both HapMap3 and MEGA array variants altered correlation patterns and the estimates remained broadly consistent (Figure 2B, Supplementary Table 5).

### Genomic enrichment of breast cancer heritability

To investigate the regulatory landscape underlying breast cancer heritability, we estimated the proportion of SNP-based heritability attributable to 73 functional genomic annotations using stratified LD-score regression^26,27^ (Methods, Supplementary Table 6). These annotations, based on the baseline-LD v2.2 model, include a curated set of coding, regulatory, and evolutionary genomic features previously shown to capture trait heritability^26–28^. Annotations definitions and data sources are described in the Methods. In the EUR sample, several annotations showed strong, statistically significant enrichment. The highest enrichment was found in ancient promoter regions (32.42-fold, p = 1.98×10^−4^), followed by transcription factor binding sites (TFBS) (5.79-fold, p = 9.16×10^−5^), and the promoter-associated histone mark H3K4me3 (4.9-fold, p = 8.56×10^−5^). We also observed significant enrichment in super-enhancers (3.01-fold, p = 3.73×10^−11^), and enhancer-associated marks H3K4me1 (2.52-fold, p = 1.28×10^−7^) and H3K27ac (2.16-fold, p = 2.25×10^−10^), highlighting the contribution of regulatory elements to genetic risk. In the EAS sample, the only annotation reaching statistical significance after Bonferroni correction (p < 6.84×10^−4^) was super-enhancers (2.73-fold, p = 2.6×10^−7^). In the AFR sample, significant enrichment was found for H3K27ac (2.15-fold, p = 2.59×10^−4^). No statistically significant enrichment was observed in the H/L sample. Patterns of enrichment for annotations that were significantly enriched in at least one ancestry sample were generally consistent across ancestries (Supplementary Figure 4). A heterogeneity test across samples for these five annotations revealed no statistically significant differences after Bonferroni correction.

### Cell-type specific associations with breast cancer risk

**Figure 3:**
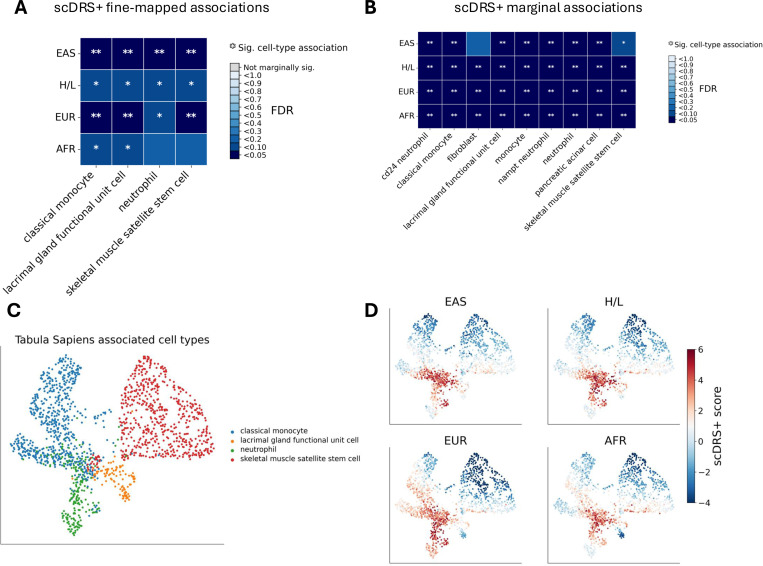
scDRS+ results on Tabula Sapiens. One asterisk indicates FDR<0.1, two asterisks indicates FDR<0.05. A) Fine-mapped associations from scDRS+, showing FDR values for cell types with at least one ancestry having FDR < 0.05; all shown associations are also marginally significant. B) Marginal associations from scDRS+, showing FDR values for cell types with associations across all traits. C) MAP of the cell types shown in panel A. D) UMAPs of scDRS+ scores across ancestries (EAS, EUR, AFR, AMR) for the same cell types as in C. Red indicates higher disease relevance.

To identify cell types associated with breast cancer risk across ancestries, we applied scDRS+^29,30^, to GWAS summary statistics from each ancestry and the Tabula Sapiens scRNA-seq atlas^31^ (133 cell types across 24 tissues). scDRS+ computes disease relevance score for individual cells based on expression of GWAS-prioritized genes, then aggregates these scores to test cell-type enrichments. For robustness, we prioritized cell types showing associations in more than three ancestry samples for robustness, reporting both fine-mapped (Figure 3A, Supplementary Table 7) and marginal results (Figure 3B; Supplementary Table 8).

First, we observed associations in several innate immune cell types, including classical monocytes (false discovery rate (FDR) <0.05 in EAS and EUR; FDR<0.1 in H/L and AFR, Figure 3A) and neutrophils (FDR<0.05 in EAS; FDR<0.1 in H/L and EUR, Figure 3A), consistent with the established role of myeloid cells in breast cancer biology and the tumor microenvironment^32–37^. Second, we detected associations for lacrimal gland functional unit cells (FDR<0.05 in EAS and EUR; FDR<0.1 in H/L and AFR, Figure 3A) and skeletal muscle satellite stem cells (FDR<0.05 in EAS and EUR; FDR<0.1 in H/L, Figure 3A). These associations are unlikely to reflect direct etiological cell types for breast cancer but may instead arise from shared epithelial or stromal regulatory programs in mammary tissue. For example, luminal epithelial cells of mammary gland were non-significant in fine-mapping but showed marginal association (FDR<0.05 in AFR; FDR≈0.07–0.10 for EAS, EUR, and H/L, Supplementary Table 8), while fibroblasts of breast were likewise non-significant in fine-mapping but displayed marginal associations in EAS (FDR=0.049), H/L (FDR=0.02), and AFR (FDR=0.008), with a weaker signal in EUR (FDR=0.11). Third, these findings are supported by consistent marginal association signals across all samples (Figure 3B), which we included to mitigate the interpretative challenges inherent in fine-mapping correlated cell populations.

To assess shared cellular signatures across ancestry samples, we examined the correlation of marginal scDRS+ association scores for the key cell types, neutrophils, classical monocytes, and skeletal muscle satellite stem cells. The association scores for these cell types were highly correlated between ancestry pairs (Supplementary Figure 5), suggesting conserved cellular mechanisms underlying genetic susceptibility. UMAP visualizations further demonstrated overlapping disease-relevant cell clusters across samples (Figure 3C, D).

## DISCUSSION

We present a compressive cross-ancestry evaluation of the genetic architecture of breast cancer using large-scale GWAS summary statistics from AFR, EAS, EUR and H/L samples. With 220,498 cases and 200,182 controls, this is the largest such evaluation to date. Our results support a largely shared polygenic architecture of breast cancer across ancestries, with consistent enrichment of common regulatory variants and convergent cellular signatures identified through single-cell analyses. While point estimates of heritability and polygenicity varied across samples, we did not detect statistically significant heterogeneity, suggesting that observed differences likely reflect sampling variation, LD structure, or reference panel choice rather than fundamental biological divergence.

Breast cancer exhibited moderate logit-scale SNP-based heritability across all groups, with the highest point estimate observed in AFR. These estimates were robust in EUR and EAS, but more sensitive to LD reference panel choice in AFR, where using sample-matched LD from AABCG controls significantly increased heritability compared to using AFR from 1000G (Supplementary Table 2). This highlights the importance of appropriate ancestry-specific LD modeling in admixed populations^38–40^. While the point estimates varied, our formal test of heterogeneity found no statistically significant differences in heritability across samples (p=0.64). These results are broadly consistent with prior reports based on subsets of our underlying data. For example, Michailidou et al. (2017)^7^, using primarily OncoArray data (a large component of our EUR sample), estimated that common variants explained ~41% of the familial relative risk, corresponding to a logit-scale heritability of approximately 0.568, closely matching our EUR estimate of 0.501. Likewise, our AFR estimate of 0.614 is comparable to the logit-scale heritability (referred to as frailty-scale) of 0.667 reported in the recent African ancestry sample breast cancer GWAS^12^, which used the same summary statistics but a different LD reference panel. While heritability has been estimated in prior studies, these have largely focused on individual ancestry groups and used varying methodologies; to our knowledge, our study provides the first systematic, cross-ancestry comparison of logit-scale SNP-based heritability using harmonized models. We focused on the logit scale, as it provides an interpretable and robust estimate of genetic variance explained by GWAS variants and connects directly to familial risk^41^, without requiring assumptions about disease prevalence.

We also observed a high degree of polygenicity across ancestry samples. The estimated number of susceptibility markers with non-zero effects in a sparse mixture model ranged from 4,446 in EAS to 8,308 in AFR. While these differences may reflect true biological variation, such as lower LD in African genomes allowing more markers to be distinguished statistically, our heterogeneity test found no significant differences in polygenicity across samples (p=0.55). Interestingly, the AFR sample also had the highest SNP-based heritability estimate, suggesting a potentially larger contribution of common variants to risk. However, PRS performance in AFR remained lower compared to EUR and EAS at comparable sample sizes^42^. This finding is consistent with our projections (Figure 1B), which demonstrate that PRS accuracy is not only a function of total heritability, but also of the effect-size distribution, i.e., the degree of polygenicity, and the LD structure of the population. Thus, the higher heritability in AFR may be offset by greater polygenicity and LD complexity, both of which reduce the efficiency of risk prediction models based on common variants.

Genetic correlations were strongest between EUR and EAS (*ρ* = 0.79), and weakest for comparison involving AFR, reflecting ancestral differences in LD structure and variant tagging. These findings may reflect known patterns of human migration and population divergence indicated from populations genetics, including an initial divergence in line with the “Out-of-Africa” migration model, with subsequent and more recent separation between European, East Asian, and American populations^43^. The moderately strong correlation between EUR and H/L (*ρ* = 0.68) likely reflects the substantial European ancestry present in many H/L individuals. However, it is important to note that this average may obscure heterogeneity within H/L populations: individuals with higher proportions of Indigenous American ancestry would be expected to show lower genetic correlation with EUR populations. The H/L summary statistics analyzed here were derived from both U.S.-based cohorts (e.g., NC-BCFR/SFBCS, RPGEH, MEC) and Latin American studies (COLUMBUS and CAMA), encompassing a mix of Mexican, Central American, and South American individuals with variable proportions of European, Indigenous American, and African ancestry. Similarly, the AFR GWAS data primarily reflect African-American individuals with admixed West African and European ancestry. As such, the estimated genetic correlations represent average effect-size sharing across ancestrally diverse groups and may mask finer-scale heterogeneity. Future analyses incorporating local ancestry or ancestry-stratified GWAS may help to disentangle these layered genetic relationships more precisely.

Stratified heritability enrichment revealed consistent contributions from regulatory elements, including promoters, enhancers, and histone marks (e.g., H3K27ac, H3K4me1), across ancestry samples. While some differences in the strength of enrichment were observed, heterogeneity tests did not identify statistically significant variation in enrichment patterns across samples. In the H/L sample, no annotations reached statistical significance, likely due to limited GWAS sample size and reduced power to detect enrichment.

Integrating GWAS with single-cell transcriptomic data via scDRS+^29^, we identified consistent enrichments in classical monocytes, neutrophils, lacrimal gland functional unit cell, and skeletal muscle satellite stem cells for breast cancer-associated gene expression across ancestries. These findings suggest that genetic risk variants may exert their effects through immune, epithelial, and stromal cells, such as inflammation, innate immune responses, or tissue repair, rather than solely within breast epithelial cells. Monocyte and neutrophil associations align with prior evidence of myeloid roles in tumor inflammation and progression ^32–37^. The lacrimal gland functional unit cells and skeletal muscle satellite stem cell associations are unlikely to reflect direct etiological cell types for breast cancer but may instead arise from shared epithelial or stromal regulatory programs in mammary tissue, such as those observed for luminal epithelial cells of mammary gland (FDR<0.05 in EAS; FDR<0.2 in EUR and AFR) and fibroblasts of breast (non-significant in fine-mapping but showing marginal association at FDR<0.05 in EAS,H/L and AFR). Alternatively, they could support emerging roles for muscle or stromal niches in metastatic reactivation ^44^. While scDRS+ does not directly identify the tissue of origin, it provides a hypothesis-generating framework that links genetic risk to specific cellular contexts based on expression profiles. The cross-ancestry consistency reinforces shared biological pathways underlying breast cancer risk in diverse populations.

This is the largest cross-ancestry investigation of breast cancer architecture; however, our study was limited by relatively small sample sizes particularly in non-European samples. Our findings highlight the value of large samples from diverse populations in breast cancer GWAS, which enables more powerful modeling of the underlying polygenic risk by leveraging similar and distinct linkage disequilibrium patterns across populations. Notably, important efforts are being made to increase samples size of breast cancer GWAS among women of Hispanic/Latina descent by the Latin American Genomics of Breast Cancer Consortium (LAGENO-BC) and among women of African descent by AABCG^12^. Moreover, the Confluence Project is building a large, diverse research resource of approximately 300,000 breast cancer cases and 300,000 controls by coordinating collaborations between LAGENO-BC, AABCG^12^, BCAC, the Consortium of Investigators of Modifiers of BRCA1/2 (CIMBA), the Male Breast Cancer Genetics Consortium (MERGE), and other breast cancer studies and clinical trials, that will help support and lead to an improved understanding of breast cancer etiology, and improvements in risk stratification and prevention across diverse populations. In summary, our study refines the understanding of breast cancer genetics across populations and highlights the value of multi-ancestry studies of breast cancer for gaining better insights in breast cancer etiology and polygenic risk prediction and stratification.

## METHODS

### Study samples and data collection

We conducted analyses using breast cancer GWAS summary statistics from large genetic consortia focused on AFR, EAS, EUR and H/L samples, with a total of 159,297 cases and 212,102 controls across large genetic consortia. Sample labels (AFR, EAS, EUR, H/L) were based on published descriptions of contributing GWAS. For AFR, data were obtained from AABCG, with 18,034 cases and 22,104 controls^12^. For EAS, we conducted a fixed-effect meta-analysis to combine the GWAS summary statistics generated from the BCAC^7^ and Biobank Japan^45^, comprising a total of 20,393 cases and 86,329 controls. For EUR, we used summary statistics from BCAC (iCOGs and OncoArray) with a combined sample size of 118,474 cases and 96,201 controls^4^. H/L ancestry summary statistics were derived from a meta-analysis of five studies^15^: NC-BCFR/SFBCS (Northern California Breast Cancer Family Registry/ San Francisco Bay Area Breast Cancer Study), RPGEH (Research Program on Genes, Environment and Health), MEC (Multiethnic cohort), COLUMBUS and CAMA (Cancer de mama) including 2,396 cases and 7,468 controls. Supplementary Table 1 summarizes the studies included in the analyses. Processing of meta-analyzed GWAS summary statistics for subsequent analysis is detailed in Supplementary Figure 1.

### Sources of genetic data for LD score construction

We constructed ancestry-specific LD score reference panels using unrelated individuals from Phase 3 of the 1000G^20^, excluding first- and second-degree relatives as well as individuals with first-cousin-level relatedness due to inbreeding^46^. For the EUR, EAS, and H/L samples, LD scores were computed using 484 EUR, 480 EAS, and 317 AMR individuals in the 1000G reference panel, respectively. For the AFR sample, we estimated LD scores using a set of 2,730 AFR women controls from the AABCG consortium. As a sensitivity analysis, we also generated LD scores using 575 AFR individuals from 1000G for comparison. The LD scores were conducted using LDSC with a LD window of 1MB using the flag “--l2 --ld-window-kb 1000”.

### Estimating ancestry-specific heritability of breast cancer

We estimated the logit-scale sample-specific SNP-based heritability for overall breast cancer risk using LD score regression^17^ and using the ancestry-sample-specific GWAS summary statistics. On the logit scale, heritability is defined as: $\sigma_{GWAS}^{2}=Var\left(\sum_{m=1}^{M}\beta_{m}G_{m}\right)$, where $G_{m}$ is the standardized genotype for the mth SNP, $\beta_{m}$ is the underlying log odds ratio, and M is the total number of causal SNPs among the GWAS variants. This quantity, also referred to as frailty-scale heritability, represents the variance in log-odds of disease explained by common genetic variants. To estimate the logit-scale heritability using LD-score regression, we computed the effective sample size as follows:

$$N_{j}={\left(2s_{j}^{2}p_{j}\left(1-p_{j}\right)\right)}^{-1}$$

where $s_{j}$ is the standard error of the log odds ratio, and $p_{j}$ is the effect allele frequency for variant j. For imputed variants, we adjusted the effective sample size to account for imputation uncertainty:

$$N_{j}=r_{j}^{2}{\left(2s_{j}^{2}p_{j}\left(1-p_{j}\right)\right)}^{-1}$$

where $r_{j}^{2}$ is the imputation quality score for variant j. To reduce the influence of poorly powered variants, we excluded those with effective sample sizes in the lowest 10% quantile and retained only the top 90% for heritability analysis. To assess the robustness of our heritability estimates, we conducted sensitivity analyses using varying SNP inclusion criteria, including: 1) using variants present in HapMap3 alone versus variants in HapMap3 plus the MEGA array, and 2) varying the MAF thresholds of between MAF > 0.01 or MAF > 0.05.

### Assessing polygenicity of breast cancer across samples

To assess the polygenicity of genetic effects of breast cancer risk within each sample, we utilized the GENESIS framework^21,22^, which models GWAS summary statistics under a three-component mixture model. Analyses were constrained to variants with MAF > 0.05 and in HapMap3 panel, following the guidance from GENESIS. For EUR and EAS samples, we used the precomputed LD provided by GENESIS. For the AFR and H/L samples, we constructed ancestry-specific LD-scores using individuals from the 1000G AMR samples, and the 2730 AA controls from AABCG, as described in the heritability analysis section. Following GENESIS’s guidance, LD scores were computed by defining tagging variants as those within a 1Mb window size and with an estimated LD coefficient $r^{2}$ above 0.1. GENESIS models the distribution of genetic marker effect sizes using a mixture of three components, including two normal distributions for non-null genetic markers with distinct variance components. This allows the model to capture both highly polygenic background signals and a subset of genetic markers with larger effect sizes. From the fitted model, we estimated the total number of susceptibility genetic markers with non-zero effect sizes. We also calculated the proportion of logit-scale heritability explained by a CT-PRS. The CT-PRS^23,24^ is a commonly used method that selects approximately independent variants through LD clumping and optimizes p-value thresholds to maximize predictive performance. Finally, we projected the expected AUC for CT-PRS at varying GWAS sample sizes based on the model estimates.

### Examining genetic correlations of breast cancer risk between samples

To estimate cross-sample genetic correlations in breast cancer risk, we utilized the Popcorn package^25^. Cross-ancestry LD reference panels were built from the same ancestry-specific sources used in heritability analyses. We applied a MAF filter >0.01 for EUR, EAS, and AFR samples, and >0.05 for the H/L sample to ensure robustness in LD estimation. We then utilized the default settings in Popcorn to compute pairwise, cross-sample genetic correlations for 1) variants in the HapMap3 reference panel and 2) the combined set of HapMap3 and MEGA array variants.

### Genomic enrichment analysis using stratified LD-score regression

We used S-LDSC to estimate the contribution of functional genomic annotations to SNP-based heritability of breast cancer^26,27^. S-LDSC models GWAS test statistics as a function of LD to variants in annotated regions, allowing partitioning of heritability while accounting for LD structure and overlapping annotations. We used 73 binary annotations from the baseline-LD v2.2 model^26–28^, which includes regulatory, coding, and evolutionary features compiled from ENCODE, Roadmap Epigenomics, and comparative genomics data. Key annotations discussed in the Results include:

1. Ancient promoters: Promoter elements whose sequence aligns to species that diverged before the placental–marsupial split, representing deeply conserved regulatory regions^28^.
2. TFBS: Genomic regions identified from aggregated ENCODE ChIP-seq^47^ and DNase I footprinting data^48^, representing sites of transcription factor occupancy.
3. H3K4me3: A histone modification marking active promoter regions, aggregated across tissues from ENCODE^47^ and Roadmap Epigenomics^49^.
4. H3K4me1: A mark associated with primed or poised enhancers^27^.
5. H3K27ac: A mark of active enhancers and promoters^27^.
6. Super-enhancers: Large regulatory domains composed of clustered enhancers with high H3K27ac signal intensity^50^.

We restricted analyses to SNPs from the HapMap3 panel with minor allele frequency >5%. For EUR and EAS, we used precomputed LD scores (https://zenodo.org/records/10515792). For AFR and H/L, we computed ancestry-specific LD scores using reference samples from the AABCG consortium (AFR) and 1000 Genomes AMR (H/L), combining annotations from EUR and EAS sources. Enrichment was defined as the proportion of heritability explained by SNPs in each annotation divided by the proportion of SNPs overlapping that annotation.

### Cell-type specific associations analysis using scDRS+

To identify cell types associated with breast cancer risk, we applied scDRS+^29,30^, an improved version of scDRS^30^ with enhanced accuracy through single-cell imputation and modeling of associations among related cell populations, to GWAS summary statistics from each ancestry alongside the Tabula Sapiens scRNA-seq atlas^31^ (27,051 cells from 133 cell types across 24 tissues; 50 cell types with >100 cells). This included 580 cells from the mammary tissue (cell types with >100 cells: luminal epithelial cells of mammary gland, T cells, fibroblast of breast), and immune cell types (cell types with >300 cells across all tissues: macrophage, CD8-positive, alpha-beta T cell, CD4-positive, alpha-beta T cell, memory B cell, monocyte, plasma cell, B cell, T cell, classical monocyte, NK cell, innate lymphoid cell, immune cell, CD8-positive, alpha-beta memory T cell, neutrophil, CD4-positive, alpha-beta memory T cell, naive thymus-derived CD4-positive, alpha-beta T cell).

To enhance data quality, we restricted analyses to fluorescence-activated cell-sorting cells. For each ancestry, we incorporated the top 1,000 MAGMA-prioritized genes^51^ into the scDRS+ framework to calculate cell-level disease relevance scores. We first identified marginally associated cells using an FDR threshold of 0.1 (analogous to scDRS), followed by joint fine-mapping to identify cells associated with breast cancer across ancestries. Cell-type-disease enrichments were tested by aggregating cell-level scores (marginal and joint) following the scDRS pipeline^30^.

To evaluate cross-sample concordance, we calculated the correlation of scDRS+ marginal scores between each pair of ancestries across all cells in a given cell type. We compared these to an empirical null distribution derived from control scores to obtain p-values for significant nonzero correlation. For all analyses, variant sets were restricted to HapMap3 + MEGA SNPs, using MAF > 0.01 for AFR, EAS, and EUR, and MAF > 0.05 for H/L.

## Supplementary Material

Supplement 1

Supplement 2

Supplement 3

## Figures and Tables

**Table 1.** Sample-specific estimates of the logit-scale SNP-based heritability

Sample	Cases	Controls	MAF cutoff	LD Score	No. of SNPs	Heritability^e^
AFR^c^	18,034	22,104	MAF > 0.01	AABCG	1,628,778	0.614 (0.095)
EAS^b^	20,393	86,329	MAF > 0.01	1000G	1,189,628	0.466 (0.066)
EUR^a^	118,474	96,201	MAF > 0.01	1000G	1,415,774	0.501 (0.050)
H/L^d^	2,396	7,468	MAF > 0.05	1000G	1,138,603	0.588 (0.360)

Summary statistics from

a the Breast Cancer Association Consortium (BCAC) (iCOGs and OncoArray)

b BCAC (OncoArray) and Biobank Japan

c Summary statistics from the African Ancestry Breast Cancer Genetic (AABCG) consortium

d NC-BCFR/SFBCS (Northern California Breast Cancer Family Registry/ San Francisco Bay Area Breast Cancer Study), RPGEH (Research project on genes environment and health), MEC (Multiethnic cohort), COLUMBUS and CAMA (Cancer de mama) studies/consortia. Variants included in the analyses were on HapMap3 + Multi-Ethnic Genotyping Array (MEGA).

e logit-scale heritability (standard error). The logit-scale heritability (also known as frality-scale heritability) is defined as $\sigma_{GWAS}^{2}=Var\left(\sum_{m=1}^{M}\beta_{m}G_{m}\right)$, where $G_{m}$ is the standardized genotype for the mth SNP, $\beta_{m}$ is the true log odds ratio for the mth SNP and M is the total number of causal SNPs among the GWAS variants. AFR, African; H/L, Hispanic and Latina; EAS, East Asian; EUR, European; MAF, minor allele frequency; LD, linkage disequilibrium; SNPs, single nucleotide polymorphisms.

## Data Availability

AFR GWAS summary statistics are available at GWAS Catalog (GCST90296719) BCAC EAS GWAS summary statistics are available at: https://www.ccge.medschl.cam.ac.uk/breast-cancer-association-consortium-bcac/data-data-access/summary-results/gwas-summary-results Biobank Japan GWAS summary statistics are available at: https://pheweb.jp/pheno/BrC BCAC EUR GWAS summary statistics are available at: https://www.ccge.medschl.cam.ac.uk/breast-cancer-association-consortium-bcac/data-data-access/summary-results/gwas-summary-associations The H/L GWAS summary statistics are available by contacting Dr. Elad Ziv (elad.ziv@ucsf.edu) to gain access.

## CODE AVAILABILITY

All data analysis code is available at: https://github.com/confluence-breast-cancer-consortia/pre-confluence-analysis/tree/main

LDSC and stratified LDSC are available at: https://github.com/bulik/ldsc

GENESIS is available at: https://github.com/yandorazhang/GENESIS

Popcorn is available at: https://github.com/brielin/Popcorn

scDRS is available at: https://github.com/martinjzhang/scDRS

## References

[1] Sung H. Global Cancer Statistics 2020: GLOBOCAN Estimates of Incidence and Mortality Worldwide for 36 Cancers in 185 Countries. CA Cancer J. Clin. 71, 209–249 (2021). 33538338 10.3322/caac.21660

[2] Jia G. Genome- and transcriptome-wide association studies of 386,000 Asian and European-ancestry women provide new insights into breast cancer genetics. Am. J. Hum. Genet. 109, 2185–2195 (2022). 36356581 10.1016/j.ajhg.2022.10.011 PMC9748250

[3] Adedokun B. Cross-ancestry GWAS meta-analysis identifies six breast cancer loci in African and European ancestry women. Nat. Commun. 12, (2021). 10.1038/s41467-021-24327-x PMC8263739 34234117

[4] Zhang H. Genome-wide association study identifies 32 novel breast cancer susceptibility loci from overall and subtype-specific analyses. Nat. Genet. 52, 572–581 (2020). 32424353 10.1038/s41588-020-0609-2 PMC7808397

[5] Michailidou K. Genome-wide association analysis of more than 120,000 individuals identifies 15 new susceptibility loci for breast cancer. Nat. Genet. 47, 373–380 (2015). 25751625 10.1038/ng.3242 PMC4549775

[6] Michailidou K. Large-scale genotyping identifies 41 new loci associated with breast cancer risk. Nat. Genet. 45, 353–361 (2013). 23535729 10.1038/ng.2563 PMC3771688

[7] Michailidou K. Association analysis identifies 65 new breast cancer risk loci. Nature 551, 92–94 (2017). 29059683 10.1038/nature24284 PMC5798588

[8] Shu X. Identification of novel breast cancer susceptibility loci in meta-analyses conducted among Asian and European descendants. Nat. Commun. 11, 1–9 (2020). 32139696 10.1038/s41467-020-15046-w PMC7057957

[9] Han M. R. Genome-wide association study in East Asians identifies two novel breast cancer susceptibility loci. Hum. Mol. Genet. 25, 3361 (2016). 27354352 10.1093/hmg/ddw164 PMC5179918

[10] Feng Y. A comprehensive examination of breast cancer risk loci in African American women. Hum. Mol. Genet. 23, 5518–5526 (2014). 24852375 10.1093/hmg/ddu252 PMC4168823

[11] Huo D. Genome-wide association studies in women of African ancestry identified 3q26.21 as a novel susceptibility locus for estrogen receptor negative breast cancer. Hum Mol Genet. 25, 4835–4846 (2016). 28171663 10.1093/hmg/ddw305 PMC5975608

[12] Jia G. Genome-wide association analyses of breast cancer in women of African ancestry identify new susceptibility loci and improve risk prediction. Nat. Genet. 56, 819– 826 (2024). 38741014 10.1038/s41588-024-01736-4 PMC11284829

[13] Hayat M. Genome-wide association study identifies common variants associated with breast cancer in South African Black women. Nat. Commun. 16, 3542 (2025). 40229280 10.1038/s41467-025-58789-0 PMC11997036

[14] Hoffman J. Identification of novel common breast cancer risk variants at the 6q25 locus among Latinas 06 Biological Sciences. Breast Cancer Res. 21, (2019). 10.1186/s13058-018-1085-9 PMC6332913 30642363

[15] Fejerman L. Genome-wide association study of breast cancer in Latinas identifies novel protective variants on 6q25. Nat. Commun. 5, 1–8 (2014). 10.1038/ncomms6260 PMC4204111 25327703

[16] Roberts E., Howell S. & Evans D. G. Polygenic risk scores and breast cancer risk prediction. Breast 67, 71–77 (2023). 36646003 10.1016/j.breast.2023.01.003 PMC9982311

[17] Bulik-Sullivan B. K. LD Score regression distinguishes confounding from polygenicity in genome-wide association studies. Nat. Genet. 47, 291–295 (2015). 25642630 10.1038/ng.3211 PMC4495769

[18] Altshuler D. M. Integrating common and rare genetic variation in diverse human populations. Nature 467, 52–58 (2010). 20811451 10.1038/nature09298 PMC3173859

[19] Bien S. A. Strategies for Enriching Variant Coverage in Candidate Disease Loci on a Multiethnic Genotyping Array. PLoS One 11, 167758 (2016). 10.1371/journal.pone.0167758 PMC5156387 27973554

[20] Auton A. A global reference for human genetic variation. Nature 526, 68–74 (2015). 26432245 10.1038/nature15393 PMC4750478

[21] Zhang Y., Qi G., Park J. H. & Chatterjee N. Estimation of complex effect-size distributions using summary-level statistics from genome-wide association studies across 32 complex traits. Nat. Genet. 50, 1318–1326 (2018). 30104760 10.1038/s41588-018-0193-x

[22] Zhang Y. D. Assessment of polygenic architecture and risk prediction based on common variants across fourteen cancers. Nat. Commun. 11, 1–13 (2020). 32620889 10.1038/s41467-020-16483-3 PMC7335068

[23] Purcell S. M. Common polygenic variation contributes to risk of schizophrenia and bipolar disorder. Nature 460, 748–752 (2009). 19571811 10.1038/nature08185 PMC3912837

[24] Wray N. R., Goddard M. E. & Visscher P. M. Prediction of individual genetic risk to disease from genome-wide association studies. Genome Res. 17, 1520–1528 (2007). 17785532 10.1101/gr.6665407 PMC1987352

[25] Brown B. C., Ye C. J., Price A. L. & Zaitlen N. Transethnic Genetic-Correlation Estimates from Summary Statistics. Am. J. Hum. Genet. 99, 76–88 (2016). 27321947 10.1016/j.ajhg.2016.05.001 PMC5005434

[26] Gazal S. Linkage disequilibrium-dependent architecture of human complex traits shows action of negative selection. Nat. Genet. 49, 1421–1427 (2017). 28892061 10.1038/ng.3954 PMC6133304

[27] Finucane H. K. Partitioning heritability by functional annotation using genome-wide association summary statistics. Nat. Genet. https://doi.org/10.1038/ng.3404 (2015) doi:10.1038/ng.3404. PMC4626285 26414678

[28] Hujoel M. L. A., Gazal S., Hormozdiari F., van de Geijn B. & Price A. L. Disease Heritability Enrichment of Regulatory Elements Is Concentrated in Elements with Ancient Sequence Age and Conserved Function across Species. Am. J. Hum. Genet. 104, 611–624 (2019). 30905396 10.1016/j.ajhg.2019.02.008 PMC6451699

[29] Turcan A., Zhang M. & Hou K. Accurate polygenic association of disease to individual cells in single-cell RNA-seq data. in Presented at: American Society of Human Genetics (ASHG) Annual Meeting (2024).

[30] Zhang M. J. Polygenic enrichment distinguishes disease associations of individual cells in single-cell RNA-seq data. Nat. Genet. 54, 1572–1580 (2022). 36050550 10.1038/s41588-022-01167-z PMC9891382

[31] Jones R. C. The Tabula Sapiens: A multiple-organ, single-cell transcriptomic atlas of humans. Science (1979) 376, (2022). 10.1126/science.abl4896 PMC9812260 35549404

[32] Zheng C. Neutrophils in triple-negative breast cancer: an underestimated player with increasingly recognized importance. Breast Cancer Res. 25, (2023). 10.1186/s13058-023-01676-7 PMC10373263 37496019

[33] Obeagu E. I. & Obeagu G. U. Exploring neutrophil functionality in breast cancer progression: A review. Medicine (United States) 103, E37654 (2024). 10.1097/MD.0000000000037654 PMC10977563 38552040

[34] Camargo S. Neutrophils physically interact with tumor cells to form a signaling niche promoting breast cancer aggressiveness. Nat. Cancer 6, (2025). 10.1038/s43018-025-00924-3 40055573

[35] Zhao Y. Neutrophils resist ferroptosis and promote breast cancer metastasis through aconitate decarboxylase 1. Cell Metab. 35, 1688–1703.e10 (2023). 37793345 10.1016/j.cmet.2023.09.004 PMC10558089

[36] Wang Y. H. Monocytes secrete CXCL7 to promote breast cancer progression. Cell Death Dis. 12, (2021). 10.1038/s41419-021-04231-4 PMC8599470 34789744

[37] Kresovich J. K. Prediagnostic Immune Cell Profiles and Breast Cancer. JAMA Netw. Open 3, (2020). 10.1001/jamanetworkopen.2019.19536 PMC6991268 31951276

[38] Hou K. Admix-kit: an integrated toolkit and pipeline for genetic analyses of admixed populations. Bioinformatics 40, (2024). 10.1093/bioinformatics/btae148 PMC10980565 38490256

[39] Zhong Y., Perera M. A. & Gamazon E. R. On Using Local Ancestry to Characterize the Genetic Architecture of Human Traits: Genetic Regulation of Gene Expression in Multiethnic or Admixed Populations. Am. J. Hum. Genet. 104, 1097–1115 (2019). 31104770 10.1016/j.ajhg.2019.04.009 PMC6562007

[40] Yang Z. Fine-mapping in admixed populations using CARMA-X, with applications to Latin American studies. Am. J. Hum. Genet. https://doi.org/10.1016/J.AJHG.2025.02.020, (2025) doi:10.1016/J.AJHG.2025.02.020,. PMC12120188 40147449

[41] Pharoah P. D. P. Polygenic susceptibility to breast cancer and implications for prevention. Nat. Genet. 31, 33–36 (2002). 11984562 10.1038/ng853

[42] Gao G. Polygenic risk scores for prediction of breast cancer risk in women of African ancestry: a cross-ancestry approach. Hum. Mol. Genet. 31, 3133–3143 (2022). 35554533 10.1093/hmg/ddac102 PMC9476624

[43] McEvoy B. P., Powell J. E., Goddard M. E. & Visscher P. M. Human population dispersal ‘Out of Africa’ estimated from linkage disequilibrium and allele frequencies of SNPs. Genome Res. 21, 821–829 (2011). 21518737 10.1101/gr.119636.110 PMC3106315

[44] Hagerling C. Immune effector monocyte–neutrophil cooperation induced by the primary tumor prevents metastatic progression of breast cancer. Proc. Natl. Acad. Sci. 116, 21704–21714 (2019). 31591235 10.1073/pnas.1907660116 PMC6815161

[45] Sakaue S. A cross-population atlas of genetic associations for 220 human phenotypes. Nat. Genet. 53, 1415–1424 (2021). 34594039 10.1038/s41588-021-00931-x PMC12208603

[46] Gazal S., Sahbatou M., Babron M. C., Genin E. & Leutenegger A. L. High level of inbreeding in final phase of 1000 Genomes Project. Sci. Rep. 5, (2015). 10.1038/srep17453 PMC4667178 26625947

[47] Dunham I. An integrated encyclopedia of DNA elements in the human genome. Nature 489, 57–74 (2012). 22955616 10.1038/nature11247 PMC3439153

[48] Trynka G. Chromatin marks identify critical cell types for fine mapping complex trait variants. Nat. Genet. 45, 124–130 (2012). 23263488 10.1038/ng.2504 PMC3826950

[49] Roadmap Epigenomics Consortium Integrative analysis of 111 reference human epigenomes. Nature 518, 317–329 (2015). 25693563 10.1038/nature14248 PMC4530010

[50] Hnisz D. Super-enhancers in the control of cell identity and disease. Cell 155, 934 (2013). 24119843 10.1016/j.cell.2013.09.053 PMC3841062

[51] de Leeuw C. A., Mooij J. M., Heskes T. & Posthuma D. MAGMA: Generalized Gene-Set Analysis of GWAS Data. PLoS COMPUT. Biol. 11, (2015). 10.1371/journal.pcbi.1004219 PMC4401657 25885710

