# Supplementary material for: Genetic and Cellular Architecture of Breast Cancer Risk in Multi-Ancestry Studies of 159,297 Cases and 212,102 Controls
Source: medRxiv. 2025 Nov 7:2025.08.20.25334075. Preprint. [Version 3] doi: 10.1101/2025.08.20.25334075 (PMC12407622; doi:10.1101/2025.08.20.25334075)

Supplementary Figure 1. Workflow for processing genome-wide association study (GWAS) summary statistics. Note: Minor allele frequency, MAF; Multi-Ethnic Genotyping Array, MEGA

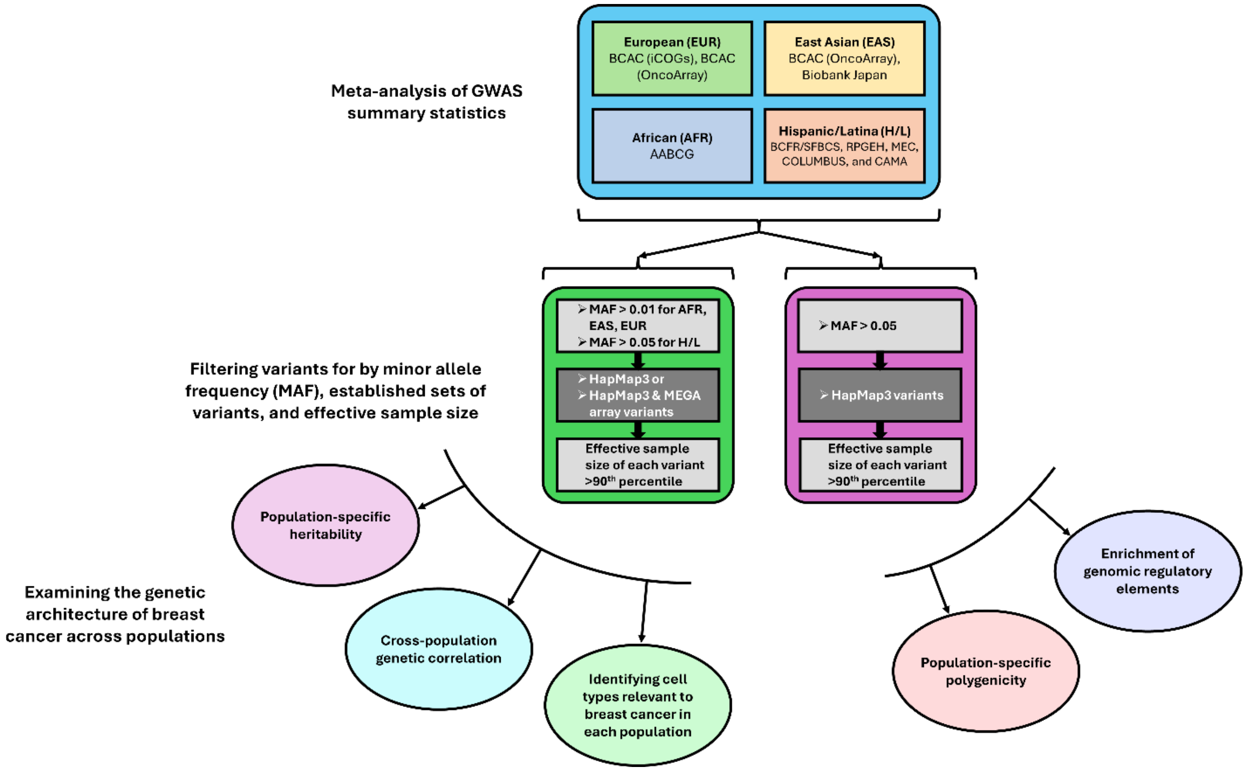

Supplementary Figure 2. Forest plot of sample-specific estimates of the logit-scale SNP-based heritability. The logit-scale heritability (also known as frailty-scale heritability) is defined as $\sigma^2_{GWAS} = Var(\sum_{m=1}^{M} \beta_m G_m)$, where G_m is the standardized genotype for the mth SNP, β_m is the true log odds ratio for the mth SNP and M is the total number of causal SNPs among the GWAS variants. AFR, African; H/L, Hispanic and Latina; EAS, East Asian; EUR, European. Error bars represent ±1 standard error. A heterogeneity test across ancestry-specific heritability estimates yielded Cochran's Q = 1.72 (p = 0.63), indicating no significant evidence of heterogeneity.

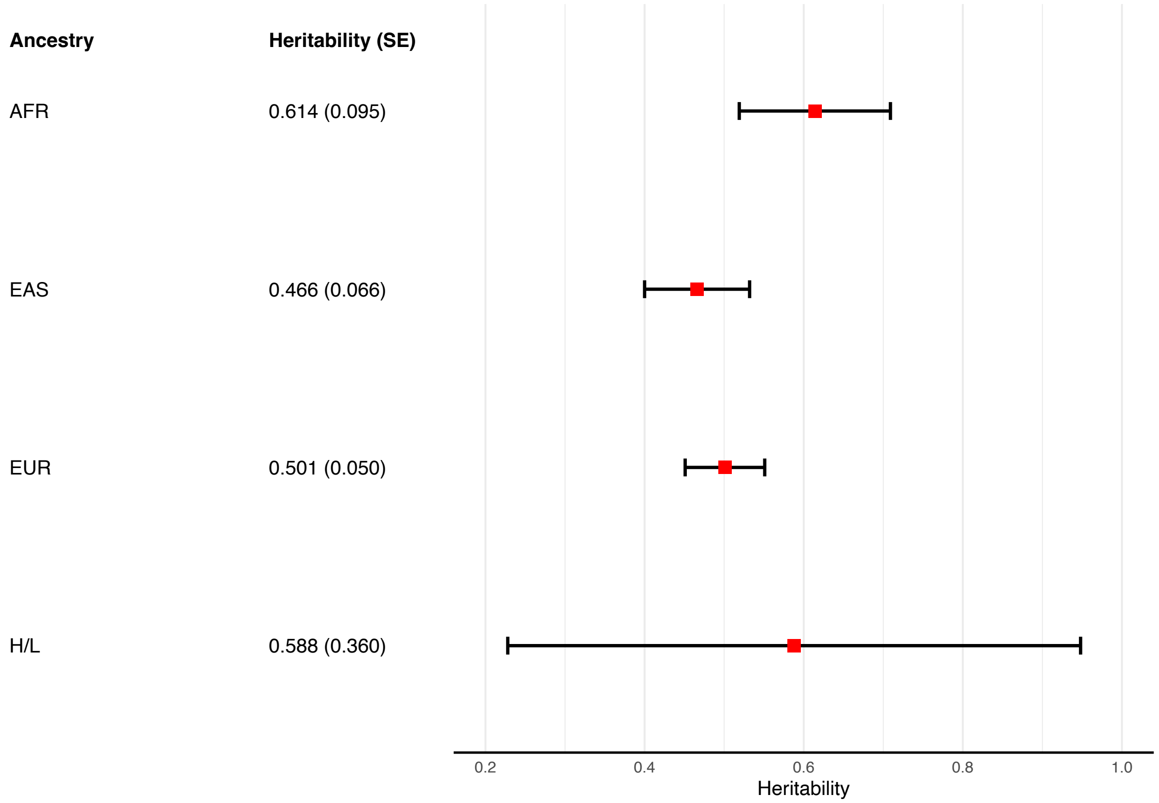

Supplementary Figure 3. Q-Q plots comparing the observed association statistic distributions and those expected under the three-component model fit by GENESIS. A) European B) East Asian C) African

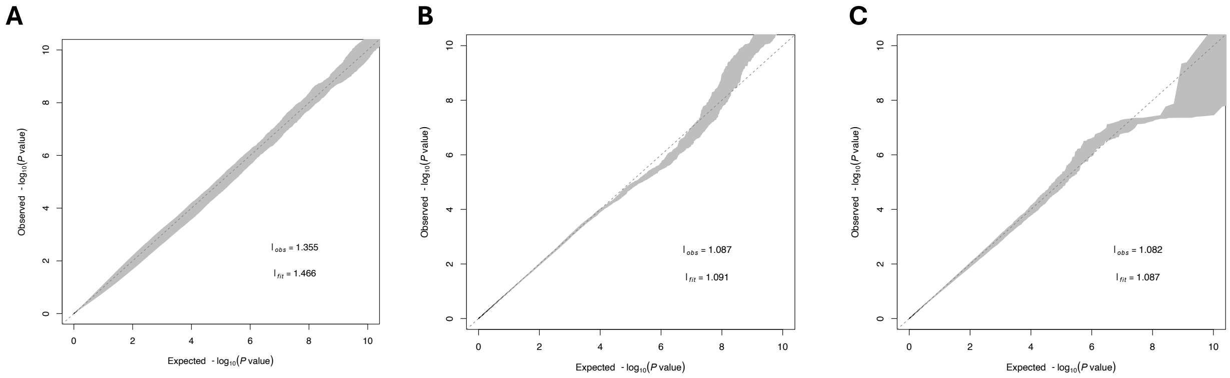

Supplementary Figure 4. Enrichment analysis results for significantly enriched annotations. Error bars represent Jackknife standard errors around the estimates of enrichment. The chi-square test was used to assess heterogeneity between ancestries. (Transcription factor for binding site (TFBS), $p = 0.82$; H3K4me3, $p = 0.483$; Super Enhancer, $p = 0.194$; H3K4me1, $p = 0.032$; H3K27ac, $p = 0.945$)

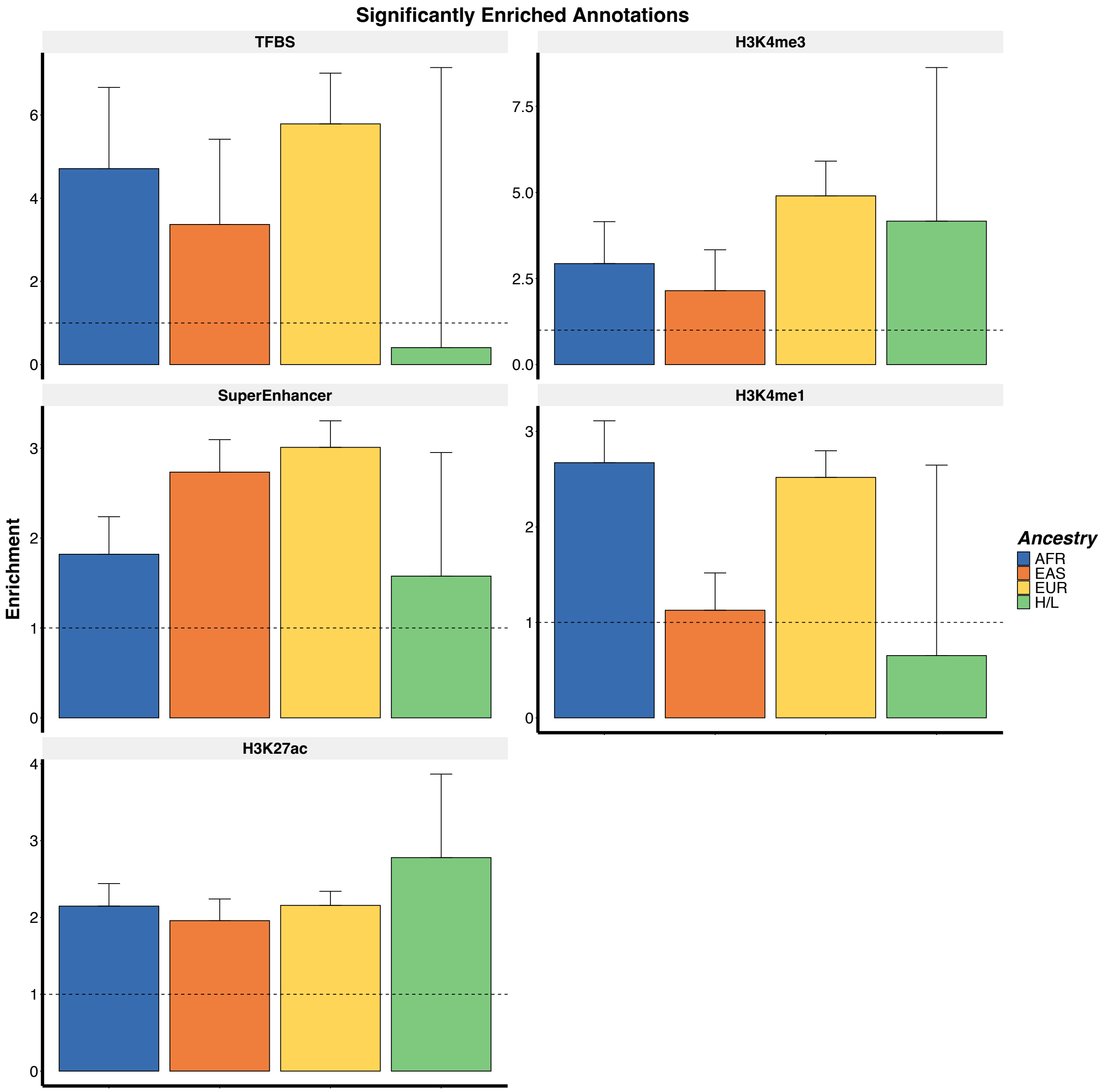

Supplementary Figure 5: **Correlation of scDRS+ scores between ancestries in A) neutrophils, B) classical monocytes, C) skeletal muscle satellite stem cells, and D) macrophages**. Significance of correlation between ancestries assessed through empirical p-values in E) neutrophils, F) classical monocytes, G) skeletal muscle satellite stem cells, and H) macrophages.

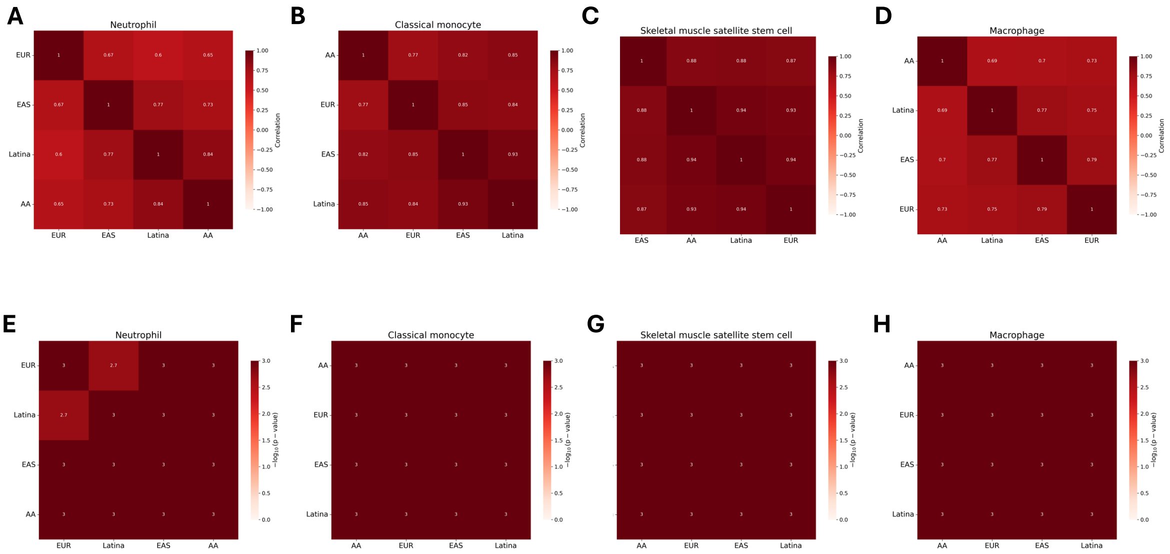

Supplement: Supplement 1 [file media-1.pdf]
