# Supplementary material for: Genetic and Cellular Architecture of Breast Cancer Risk in Multi-Ancestry Studies of 159,297 Cases and 212,102 Controls
Source: medRxiv. 2025 Nov 7:2025.08.20.25334075. Preprint. [Version 3] doi: 10.1101/2025.08.20.25334075 (PMC12407622; doi:10.1101/2025.08.20.25334075)
Supplement: Supplement 3 [file media-3.pdf]

Supplementary Acknowledgement

We would like to thank the following members of COLUMBUS Consortium:

University of California at Davis: Ana Estrada-Florez, Paul Lott, Guadalupe Polanco-Echeverry, Luis Carvajal-Carmona.

Colombia: Juan Manuel Acosta (Universidad del Tolima, Ibagué), Jennyfer Benavides (Universidad del Tolima, Ibagué), Mabel Bohorquez (Universidad del Tolima, Ibagué), Jenny Carmona (Dinámica IPS, Medellín), Ángel Criollo (Universidad del Tolima, Ibagué), Magdalena Echeverry (Universidad del Tolima, Ibagué), Ana Estrada-Florez (Universidad del Tolima, Ibagué), Gilbert Mateus (Hospital Federico Lleras Acosta, Ibagué), Raúl Murillo (Pontificia Universidad Javeriana, Bogotá,), Justo Ramirez (Hospital Hernando Moncaleano Perdomo, Neiva), Carolina Sanabria (Instituto Nacional de Cancerología, Bogotá), Yesid Sánchez (Universidad del Tolima, Ibagué), Martha Lucia Serrano (Instituto Nacional de Cancerología, Bogotá), John Jairo Suarez (Universidad del Tolima, Ibagué), Alejandro Vélez (Dinámica IPS, Medellín, Colombia, Hospital Pablo Tobón Uribe, Medellín).

Mexico: Javier Torres (Mexican Social Security Hospital).
